# A Simple and Green Procedure for the Synthesis of 5-Arylidenerhodanines Catalyzed by Diammonium Hydrogen Phosphate in Water

**DOI:** 10.1155/2013/543768

**Published:** 2013-10-22

**Authors:** Liangliang Han, Tao Wu, Zhongqiang Zhou

**Affiliations:** Key Laboratory of Catalysis and Materials Science of the State Ethnic Affairs Commission and Ministry of Education, College of Chemistry and Materials Science, South-Central University for Nationalities, Wuhan, Hubei 430074, China

## Abstract

A simple and efficient procedure for the synthesis of 5-arylidenerhodanines by condensation of aromatic aldehydes with rhodanine in water using diammonium hydrogen phosphate as catalyst is described. The procedure offers several advantages including environmentally friendly, mild reaction conditions, short reaction times, high yields, and simple experimental and work-up procedures.

## 1. Introduction

Rhodanine derivatives, especially arylidenerhodanines, have shown a wide range of pharmacological activities, which include anticonvulsant, antibacterial, antiviral, and antidiabetic effects [1–3]. Arylidenerhodanines are generally prepared by reacting aldehydes and rhodanine in organic solvents and in the presence of organic bases like piperidine [2, 4–8]. Recently, catalysts such as 2,2,6,6-tetramethyl piperidine [9, 10], NH_4_Cl/NH_4_OH [11], K_2_CO_3_/[bmim]BF_4_/H_2_O [12], NaOAc/HOAc [3, 13, 14], glycine [15], ammonium acetate [16, 17], 1-butyl-3-methyl imidazolium hydroxide [18, 19], tetrabutylammonium bromide [20], and K_2_CO_3_/Al_2_O_3_ [21] have been used in this reaction. Although these methods are valuable, most of them suffer from some disadvantages such as a long reaction time, low yields, the use of toxic solvent, expensive catalyst, requiring a promoter, such as microwave, and tedious work-up procedures. Thus, the development of a new procedure for the synthesis of arylidenerhodanines would be highly desirable.

**Scheme 1:**
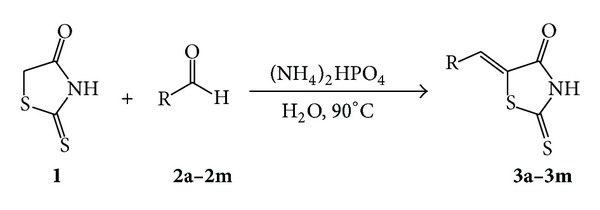
Diammonium hydrogen phosphate catalyzed synthesis of 5-arylidenerhodanines.

Water is abundant, inexpensive, safe, and clean. Among various solvents, water is the most preferred solvent. The use of water as a solvent is the strategy commonly used toward greener chemistry. A wide range of reactions that can be conducted in or on water have been developed [22–24]. Diammonium hydrogen phosphate has been used as an efficient, nontoxic, and cheap catalyst in organic synthesis [25–28]. As a part of our endeavors towards the development of efficient, and environmentally benign synthetic methodologies in water [29–32], we report herein a simple, efficient, and practical method for the synthesis of 5-arylidenerhodanines by the condensation of rhodanine with aromatic aldehydes in the presence of diammonium hydrogen phosphate in water (Scheme 1).

## 2. Results and Discussion

In order to get the best experimental reaction conditions, the reaction of rhodanine **1** and 2,4-dichlorobenzaldehyde **2a** in the presence of 10 mol% of diammonium hydrogen phosphate in water has been considered as a standard model reaction. Effects of reaction temperature on the yields of the product were studied by performing the model reaction at 80°C, 90°C, and 100°C, respectively (Table 1, entries 1–3). The yield of product **3a** was increased as the reaction was raised from 80 to 90°C. However, no increase in the yield of product **3a** was observed as the reaction temperature was raised from 90 to 100°C (Table 1, entries 2-3). Therefore, 90°C was chosen as the reaction temperature for all further reactions.

Moreover, we found that the yields were obviously affected by the amount of diammonium hydrogen phosphate loaded. When the amount of the catalyst decreased to 5 mol% from 10 mol% relative to the substrates, the yield of product **3a** was reduced (Table 1, entries 2 and 5). However, the use of 20 mol% of the catalyst showed the same yield and the same time was required (Table 1, entry 6). So, the use of 10 mol% of catalyst is sufficient to push the reaction forward. It is noteworthy that, in the absence of a catalyst under the reaction conditions, no product formation was observed after 60 min (Table 1, entry 4). This result indicates that the catalyst exhibits a high catalytic activity in this transformation.

Using these optimized reaction conditions, the scope and efficiency of this approach were explored for the synthesis of a wide variety of 5-arylidenerhodanines and the obtained results are summarized in Table 2. The reaction worked well with a variety of aldehydes including those bearing an electron-withdrawing group and electron-donating group and the corresponding products were obtained with high yields in short times.

**Scheme 2:**
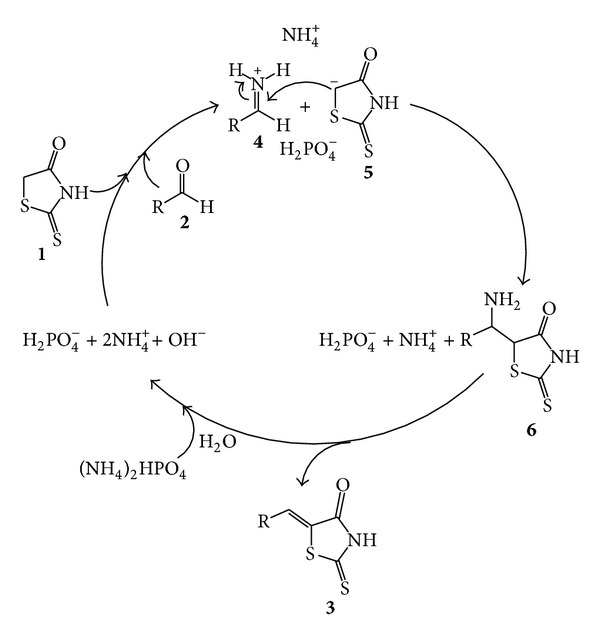
Plausible mechanism for the synthesis of 5-arylidenerhodanines catalyzed by diammonium hydrogen phosphate.

A plausible mechanism for this reaction has been suggested in Scheme 2. Ionization of diammonium hydrogen phosphate leads to the formation of hydroxide ion and ammonium ion. Subsequent reaction between the hydroxide ion and rhodanine gives rise to a rhodanine anion **5**. Meanwhile, aldehyde can form iminium ion **4** [26]. The iminium ion **4** condenses with rhodanine anion **5** to form intermediate **6**, which could be converted to 5-arylidenerhodanines **3** after elimination of ammonia.

## 3. Conclusion

In summary, a simple, efficient, and green procedure has been developed for the synthesis of 5-arylidenerhodanines in water by the condensation of rhodanine with aldehydes in the presence of diammonium hydrogen phosphate. This method provides a simple and efficient protocol in terms of environmentally friendly, mild reaction conditions, short reaction times, high yields, and simple experimental and work-up procedures.

## 4. Experimental 

### 4.1. Materials and Instrumentation

All chemicals were commercially available and were used as received. Melting points were determined on a X-4 micromelting point apparatus and are uncorrected. FT-IR spectra were obtained on a Nexus 470 spectrophotometer. ^1^H NMR spectra were recorded on a Bruker Avance III 400 with TMS as internal standard.

### 4.2. General Procedure for the Preparation of 5-Arylidenerhodanines

A mixture of rhodanine (333 mg, 2.5 mmol), the aldehyde (2.5 mmol), and diammonium hydrogen phosphate (33 mg, 0.25 mmol) in H_2_O (3 mL) was stirred at 90°C. The progress of the reaction was monitored by thin-layer chromatography (ethyl acetate : petroleum ether 1 : 1 (v : v) as eluent). After completion of the reaction, the solid material was filtered and washed with water. Further purification was carried out by crystallization from ethanol. Products obtained are all known compounds and were identified by comparing their physical and spectra data with the reported ones.

## Figures and Tables

**Table 1:** Effect of different reaction conditions on synthesis of 5-arylidenerhodanines^a^.

Entry	(NH_4_)_2_HPO_4_ (mol%)	Temperature (°C)	Time (min)	Yield (%)^b^
1	10	80	45	85
2	10	90	18	86
3	10	100	18	86
4	0	90	60	0
5	5	90	18	75
6	20	90	18	86

^a^Reaction condition: 2,4-dichlorobenzaldehyde (2.5 mmol), rhodanine (2.5 mmol), and water (3 mL).

^b^Isolated yield.

**Table 2:** Diammonium hydrogen phosphate catalyzed synthesis of 5-arylidenerhodanines^a^.

Entry	R	Time (min)	Product	Yield (%)^b^	Mp (°C) found	Mp (°C) reported
1	2,4-Cl_2_C_6_H_3_	18	**3a**	86	233–235	233-234 [12]
2	4-CH_3_C_6_H_4_	14	**3b**	85	222–224	221–223 [12]
3	4-CH_3_OC_6_H_4_	17	**3c**	88	248–250	249-250 [12]
4	2-ClC_6_H_4_	10	**3d**	88	180-181	181-182 [12]
5	4-FC_6_H_4_	9	**3e**	80	218-219	219 [1]
6	4-HOC_6_H_4_	13	**3f**	83	308–310	310 [16]
7	4-BrC_6_H_4_	15	**3g**	82	228–230	230 [1]
8	3-NO_2_C_6_H_4_	16	**3h**	90	263–265	263–265 [12]
9	4-ClC_6_H_4_	13	**3i**	81	228–230	229-230 [12]
10	C_6_H_5_	8	**3j**	86	204–206	205–207 [12]
11	2-HOC_6_H_4_	16	**3k**	84	222-223	221-222 [21]
12	2-Furyl	4	**3l**	85	227–229	228-229 [12]
13	4-HO-3-CH_3_OC_6_H_3_	11	**3m**	84	231-232	231–231.5 [21]

^a^Reaction condition: aldehyde (2.5 mmol), rhodanine (2.5 mmol), (NH_4_)_2_HPO_4_ (0.25 mmol), 90°C, and water (3 mL).

^b^Isolated yield.
